# Descemet’s membrane endothelial keratoplasty for primary graft failure after penetrating keratoplasty

**DOI:** 10.5935/0004-2749.2021-0495

**Published:** 2022-10-19

**Authors:** Bruno Lovaglio Cançado Trindade, Fellype Borges de Oliveira, Letícia Arriel Crepaldi

**Affiliations:** 1 Departament of Cornea, Instituto de Olhos, Hospital de Universitário de Ciências Médicas, Belo Horizonte, MG, Brazil; 2 Faculdade de Ciências Médicas de Minas Gerais, Belo Horizonte, MG, Brazil

**Keywords:** Corneal diseases, Corneal transplantation/adverse effects, Graft Rejection, Keratoplasty, penetrating, Descemet membrane, Descemet stripping endothelial keratoplasty, Eye infections, viral, Humans, Case reports, Doenças da córnea, Transplante da córnea/efeitos adversos, Rejeição do enxerto, Ceratoplastia penetrante, Lâmina limitante posterior, Ceratoplastia endotelial com remoção da lâmina limitante posterior, Infecções oculares virais, Humanos, Relatos de casos

## Abstract

Primary graft failure (PGF) is a known complication following penetrating keratoplasty (PKP). The usual approach to treat this complication is to repeat a penetrating keratoplasty. Here, we report a case of Descemet’s membrane endothelial keratoplasty (DMEK) for the treatment of PGF after PKP. A patient that underwent PKP, developed PGF with persistent graft edema and very poor visual acuity despite aggressive steroid use and a proof anti-viral treatment. Three months after the initial surgery, a DMEK was performed under the PKP graft. There was progressive early corneal clearing and, by the end of the first month, the patient already had no corneal edema. Uncorrected visual acuity (UCVA) improved to 20/40 and best corrected visual acuity (BCVA) to 20/20. DMEK may be an alternative to a second PKP for the treatment of PGF. This technique is a less invasive option when compared to the standard PKP procedure.

## Abstract (pt)

A falência primária do enxerto é uma complicação conhecida que pode ocorrer após o transplante penetrante de córnea. O tratamento usual dessa complicação é com um novo transplante penetrante. Apresentamos um caso em que foi usado o transplante endotelial de membrana de Descemet (DMEK - do inglês *Descemet membrane endo-thelial keratoplasty*) para o tratamento da falência primária após o transplante penetrante. Uma paciente submetida a transplante penetrante evoluiu com falência primária do enxerto a despeito do uso intenso de corticoide tópico e uma prova terapêutica de antivirais. Três meses após a cirurgia inicial, foi optado pela realização do transplante endotelial de membrana de Descemet sob o transplante penetrante. Houve um clareamento precoce e progressivo do enxerto com melhora importante da visão. Após um mês, a visão sem correção era de 20/40 melhorando para 20/20 com refração. O transplante endotelial de membrana de Descemet pode ser uma alternativa a um novo transplante penetrante como tratamento da falência primária.

## INTRODUCTION

Penetrating keratoplasty (PKP) is an established technique used in the treatment of several corneal diseases^(1)^. The incidence of primary graft failure (PGF) after PKP varies between 0 and 12%^(2)^. Primary graft failure can be defined as (1) the presence of a diffusely edematous corneal graft on the first postoperative day, (2) failure of the cloudy graft to clear at any time postoperatively and (3) lack of an identifiable cause of corneal graft failure within 2 weeks of transplantation^(3)^.

Several studies have already demonstrated the efficacy of treating late PKP graft failure with endothelial keratoplasty and, more specific, with DMEK^(4,5,6)^. This approach enables a safer procedure, faster visual rehabilitation and less chance of endothelial rejection. The purpose of this paper is to present a case a primarily failed PKP treated with DMEK.

## CASE REPORT

**Figure 1:**
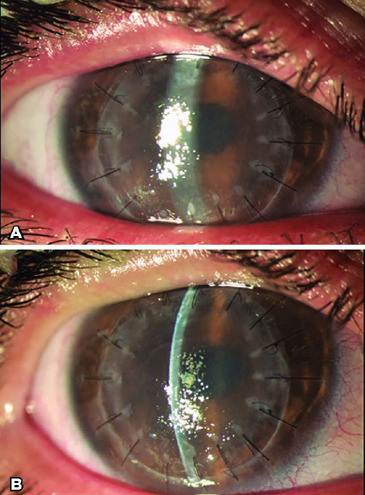
(A and B) Three months after penetrating keratoplasty. Note the persistent difuse graft edema.

A 27-year-old female underwent an uneventful PKP for advanced keratoconus. The donor cornea had an endothelial cell count of 2403 cells/mm² and had been preserved in Optisol-GS for 12 days. One week later, the graft maintained intense edema and visual acuity was worse than 20/800. Central corneal thickness was 785 µm. As per the eye bank, the contralateral do nor cornea had been successfully transplanted with slightly shorter preservation interval. An empirical treatment for Herpes simplex keratitis (HSK) was instituted with acyclovir 400 mg five-times-a-day with no improvement. With no clearing of the corneal edema, three months after the PKP, the patient underwent a phakic-DMEK for the treatment of primary graft failure. Figure 1A and B shows the aspect of the eye 3 months after PKP and before DMEK.

**Figure 2:**
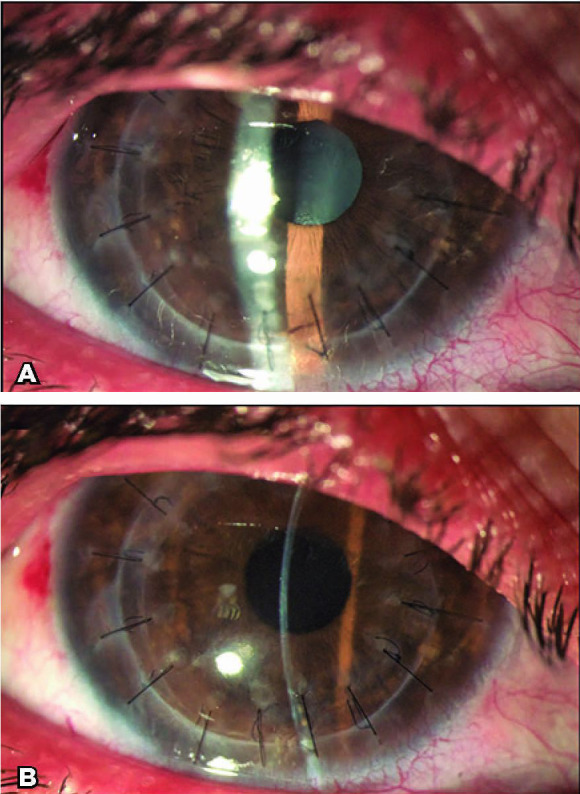
(A and B) One month after Descemet membrane endothelial keratoplasty. The patient showed a much clearer graft with improved vision.

A 54-year-old donor cornea was selected with an endothelial count of 2583 cells/mm². Descemetorhexis was performed under air with care not to traction and disrupt the graft-host-junction. A 7.0mm graft (1mm smaller than the original PKP graft) was used and was secured in place using filtered air. Because the eye was phakic and there was a significant cornea edema preoperativelly (which hindered the use of nd:YAG laser) we did not do a peripheral iridectomy and decreased the air bubble to approximately 50% to avoid pupillary block at the end of surgery. Despite being an early reoperation, no additional difficulties were encountered. During surgery, an anterior chamber tap was performed for viral PCR and came back negative for herpes simplex virus (HSV), varicella-zoster virus (VZV), cytomegalovirus (CMV) and Epstein-Barr virus (EBV). Due to internal hospital handling issues, the excised Descemet membrane was not sent to pathology and no information regarding the failed endothelium was obtained. One month after surgery, UCVA was 20/40 improving to 20/20 with -0,50 x -2,25 x 70º (Figure 2).

## DISCUSSION

Primary graft failure results from graft endothelial dysfunction that leads to persistent corneal edema present since the first days after transplantation. Important risk factors for PGF are corneal preservation time greater than seven days^(7)^, donor endothelial cell damage during preservation or storage and surgical trauma. It is important to rule out other causes of graft edema such as viral infections by HSV or CMV^(8)^.Traditionally, primary PKP graft failure is treated with a repeat PKP. Nevertheless, this approach results in inherent surgical risks of another open-sky procedure, corneal damage associated with new multiple sutures as well as the cumulative immune exposure and consequently decreased graft survival.

It has been shown that endothelial keratoplasty (EK) can be performed under a failed full-thickness graft^(4,9)^. This approach decreases the intraoperative risks and may improve the postoperative results. Both DSAEK and DMEK have been shown to be useful in this scenario. Despite being more technically challenging, DMEK may provide a faster visual rehabilitation and, more importantly in these cases, less induced endothelial rejection^(9)^.

DMEK provides a perfect substitution of the posterior layers of the cornea and has little influence on refraction and host corneal topography. Therefore, refractive outcome after DMEK under a failed PKP can be predicted based on pre-failure assessment. Any visual limitation due to opacities or irregular astigmatism present before failure will likely persist limiting the final outcome. The use of small aperture devices has been shown to successfully treat irregular corneal astigmatism and enable performing DMEK for a failed irregular PKP graft^(10)^.

However, the benefit of endothelial keratoplasty might not justify the visual limitations of the initial corneal graft and weighing the pros and cons should always be done to decide the best approach for each case. In cases of primary PKP graft failure, the final graft visual acuity is not available, and this decision is even less clear. The final graft topography can, at best, be estimated and the impact on visual acuity has to be guessed. However, it is important to highlight that, with endothelial keratoplasty under a failed-PKP, another PKP can always be performed later in case it is needed. Thus, an EK can be tried before even when the expected outcome is not completely clear.

Selective graft suture removal is expected to be performed in this scenario sometime after the endothelial keratoplasty. This may interfere with the EK graft attachment and cause a late graft detachment. However, we do not anticipate this since these grafts are usually very well adherent and the sutures are usually partial-thickness only.

To the authors’ best knowledge, there is no study evaluating the benefits of using DMEK grafts to treat a primary failed PKP and it is important to highlight that DMEK may have a relatively high incidence of primary graft failure by itself.

Endothelial keratoplasty has revolutionized corneal transplantation in the past few decades and it may significantly change the way primary failed PKP grafts are managed. Further prospective studies are needed to assess the validity of this approach in similar cases.
